# A Bibliometric History of the Journal *GENETICS*

**DOI:** 10.1534/genetics.116.196964

**Published:** 2016-10-17

**Authors:** Natalie Telis, Benjamin V. Lehmann, Marcus W. Feldman, Jonathan K. Pritchard

**Affiliations:** * Biomedical Informatics, Stanford School of Medicine, Stanford, California 94305; † Howard Hughes Medical Institute, Chevy Chase, Maryland 20815; ‡ Department of Biology, Stanford University, Stanford, California 94305; § Department of Genetics, Stanford University, Stanford, California 94305

**Keywords:** bibliometrics, history, population genetics, organism biology, genomics

Advances in genetic technology and biological understanding in the last 100 years have opened a new era of discovery and investigation. In the centennial year of the journal *GENETICS*, we quantify the remarkable shifts in genetic research through textual analysis of publications. We characterize changes in the focus of genetic research by studying all available abstracts and titles of papers published since 1916 in *GENETICS*. We document a massive expansion in publications in genetics, beginning in the 1950s, and accelerating in the 1980s, as genetic research expanded globally from a few initial locations. We also describe changes in word usage over time, reflecting evolving research interests, methods, and organisms. For example, we observe stable use of *Drosophila* in genetics research throughout the century. In contrast, we document rapid increases in human genetic research and extreme expansion in use of model organisms, including *Escherichia coli*, *Arabidopsis thaliana*, *Caenorhabditis elegans*, and *Saccharomyces cerevisiae*, and a later decline in use of prokaryotes. We use bibliometric analyses to measure the most prominent research trends as reflected in the journal *GENETICS*.

THIS year, we celebrate a century since the first issue of *GENETICS* was published. This century has come with incredible breakthroughs and growth in our understanding of genetics, and *GENETICS* has played an important role in these discoveries. Early discoveries about the mechanics and statistics of inheritance (Morgan *et al.* 1915; Wright 1931) began to uncloak the mechanisms behind heritability. The ability to begin manipulating traits—through induction of mutation—paved the way for completely novel experimentation (Muller 1928).

The next breakthrough came soon after, with identification of DNA as the crucial molecule inherited from generation to generation (Avery *et al.* 1944). This was followed by geneticists’ new ability to understand and manipulate DNA itself. Its structure was identified (Watson and Crick 1953). Variation and polymorphisms were found and catalogued (Harris 1966; Hubby and Lewontin 1966; Lewontin and Hubby 1966). An organisms’ DNA was shown to be not static—in fact, the DNA content of one organism during its lifetime might be manipulated (Luria and Delbruck 1943). Muller’s prophetic prediction—that all geneticists might also become bacteriologists (Dove 1997)—came true, and this ushered in a new age of discovery and methodology. And of course, within a few years, the ability to directly read DNA sequence illuminated and transformed genetics once again (Sanger *et al.* 1977).

Over the century, the journal *GENETICS* has been not just witness to but the birthplace of many of the major shifts in conceptual and experimental genetics. Its content provides a comprehensive view into many intellectual developments of the last 100 years. With the advent of online repositories of publications, the evolution of the field has been rendered accessible to text-analytic methods. On this centennial, we consider the publications of *GENETICS* retrospectively, through the lens of text-based computational analysis.

We use computational techniques for textual analysis of *GENETICS* to quantify its role in the groundbreaking changes in genetics over the last century. We first investigate broad trends in publication metadata and geography and present results concerning global expansion in genetics. We then investigate trends in specific word groups (delineating subfields, methodologies, and organisms studied), as well as broader decade-specific shifts that reflect the rapid growth and change in genetics.

## Summary Statistics

### Publication numbers and growth

**Figure 1:**
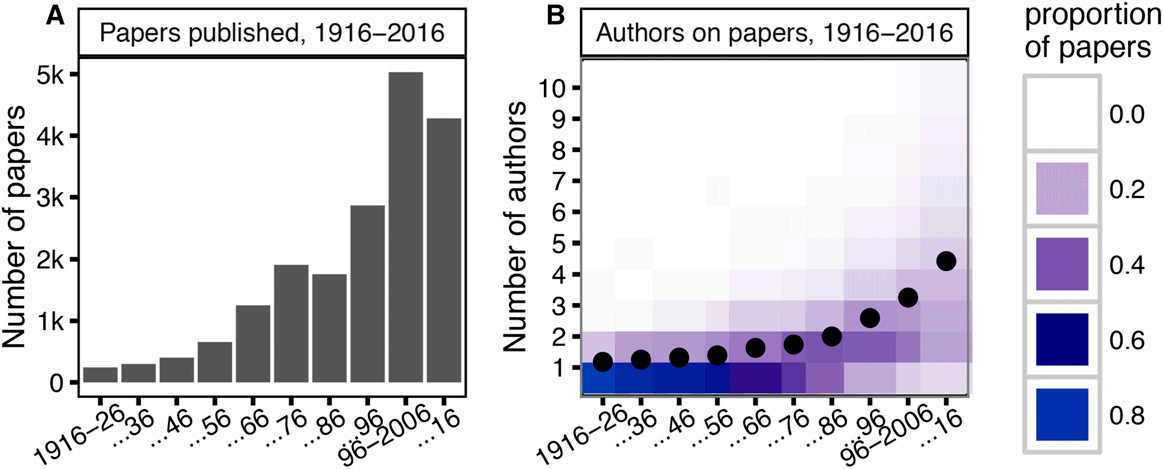
(A) The number of papers published in *GENETICS* every decade (10-year period) since its creation in 1916. (B) A heatmap of the proportions of papers per decade with any given number of authors. Proportions normalized per decade, with mean author count indicated by the overlaid black dot.

We analyzed all titles and abstracts published in *GENETICS* prior to June 7, 2016: a total of 18,698 papers. Of these, almost half were published in the last 20 years (Figure 1A). There has been a simultaneous increase in the number of authors per paper, with the mean in 2006–2016 slightly above four (compared to just one author in 1916–1926) (Figure 1B). The journal’s growth accelerated in the 1950s, when the number of papers in *GENETICS* approximately doubled. Though there was a brief plateau, the same rate of expansion recurred between the 1980s and 1990s (Figure 1A).

### Geographic distribution

**Figure 2:**
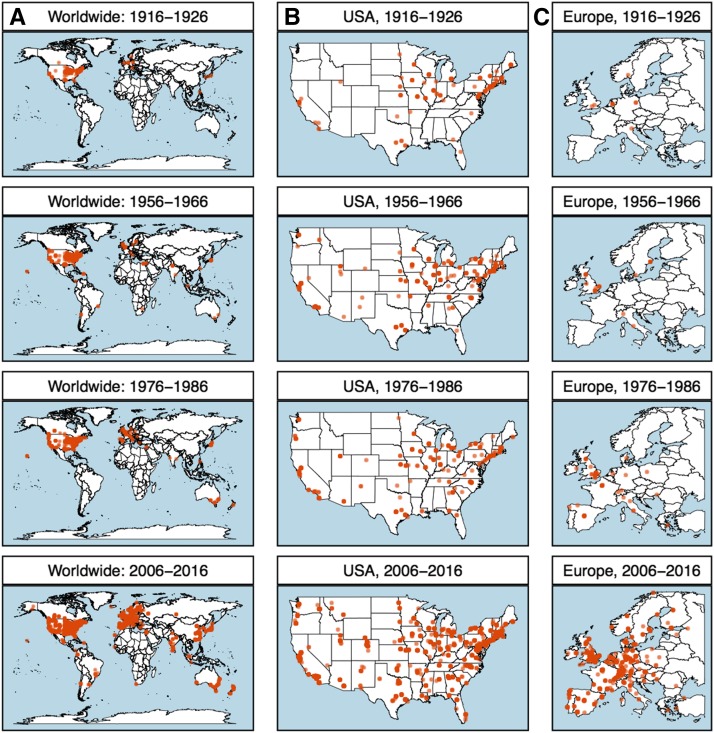
(A) A worldwide map of geographic locations of authors publishing in *GENETICS* for selected decades. Within each 10-year period depicted, each dot on the map represents the latitude and longitude of a unique affiliation of a paper published within those years. The intensity of color indicates frequency of publication from a particular location, *e.g.*, one university or department. Selected decades show significant expansion in the global reaches of science, as well as local trends. Other maps zoom in on selected decades in (B) the continental United States and (C) Europe.

The geographic distribution of authors has shifted over time, reflecting country- and world-wide economic and political trends. The journal’s early years were dominated by small regions of the United States, such as New England, with a high density of academic institutions (Figure 2B). Changes reflected the dynamics of research institutions and the corresponding landscape of working geneticists, and this landscape altered substantially throughout the century.

Expansion of the journal in the decades following World War II was especially significant in the United States (Figure 2B). Several events specific to the United States may have affected the expansion of academic publishing during this period. For example, the National Science Foundation Act of 1950 established a significant novel source of funding. Moreover, major shifts in the structure of National Institutes of Health funding established separate study sections to fund genetics, beginning in 1958 (Crow and Owen 2000). The Servicemen’s Readjustment Act of 1944 (popularly known as the “G.I. Bill”), which provided tuition for veterans, drastically increased university student enrollment (Adams 2000). After a period of stability in the number of papers published, a new expansion occurred with worldwide effects (Figure 2, A and C). This suggests influence of more global factors in the most recent expansion of *GENETICS*.

## Textual Analysis

The expansion and globalization of publication in *GENETICS* may be related to the shifts in methodology and the availability of data, so we analyzed textual trends. An analysis of all available titles and abstracts by decade yielded insights into the dynamics of genetic research across the decades.

**Figure 3:**
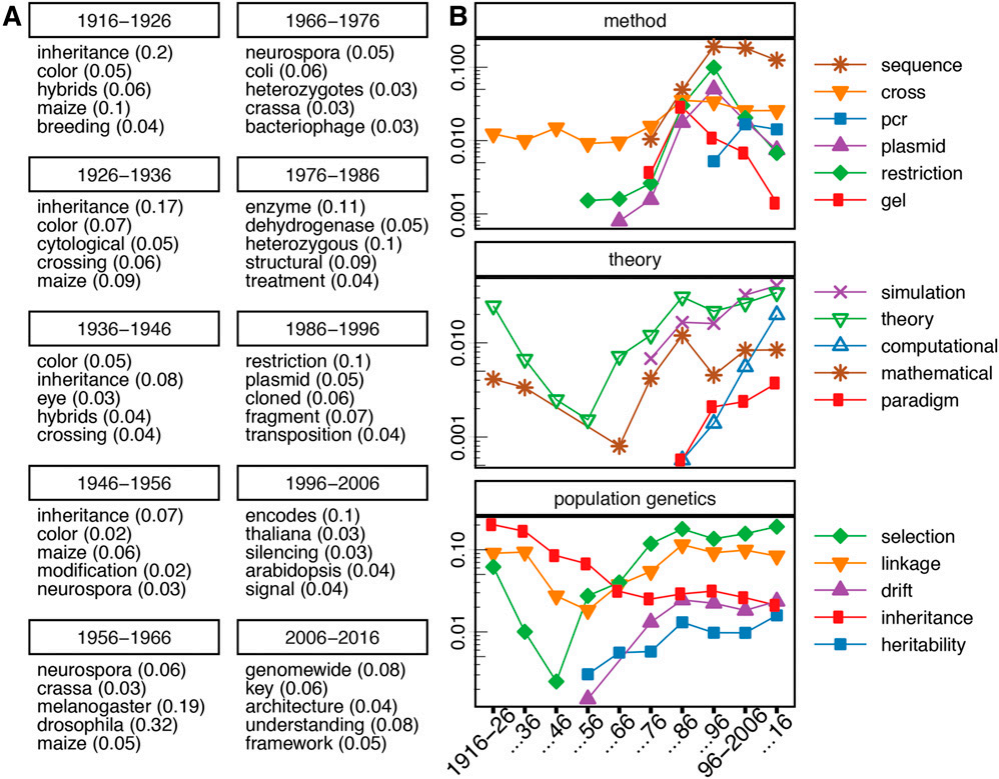
(A) Top five most enriched words per decade in all available text (titles and abstracts), displayed in order of enrichment from top to bottom, as “word (proportion)” where proportion is the proportion of all papers published that decade, which mention this word. (B) In order from top to bottom: trajectories of word use, visualized in changes in proportions of paper mentioning a word at least once. These proportions are displayed per 10-year-period for related sets of words: (i) experimental methods (method), (ii) theoretical methods (theory), and (iii) words associated with long-standing concepts in the subfield of population genetics.

Many prominent trends relate to changes in organisms studied, which are strongly linked to contemporary lines of inquiry. For example, in the journal’s first decade, 1916–1926, investigation of plant and animal phenotypes (*e.g.*, color, maize in Figure 3A) and their inheritance predominated, along with the methods involved (*e.g.*, animal and plant breeding, Figure 3A).

As microorganisms became a primary focus of research, there was a strong shift in the popularity of different model organisms. By the 1950s, microorganisms such as *Neurospora crassa* dominated (Figure 3A). This was reflected in and perhaps driven by major discoveries at the time (Beadle and Tatum 1941). A final shift between 1996 and 2006 brought *Arabidopsis thaliana* to the forefront, reflecting the advent of plant genomics (Figure 3A). Technological shifts substantially changed the vocabulary of publication in the second half of the 20th century. The identification of DNA as the molecule of inheritance (Avery *et al.* 1944) initiated a period of rapid discovery. Similarly, the identification of other mechanisms of DNA transfer (Lederberg *et al.* 1952) set in motion the development of techniques for transformation. Later, the first assays to distinguish polymorphisms by gel electrophoresis proved crucial to cataloging the extent and nature of variation (Harris 1966; Hubby and Lewontin 1966; Lewontin and Hubby 1966). Each of these historic shifts is reflected in the decade-specific enrichment of associated words such as gel, restriction, and plasmid (Figure 3B).

The enriched words that reflect a change in techniques in the last decades coincide with the advent of the genomic era and associated areas of research. Silencing appears enriched for the first time in 1996–2006, after the identification of several separate instances of transcriptional repression in yeast (Brand *et al.* 1985; Gottschling *et al.* 1990). In 2006–2016, the word “genome-wide” heads the list as the most decade specific.

Notably, some enriched words in *GENETICS* from the most recent decade are not techniques, but rather adjectives (*e.g.*, key, Figure 3A), reflecting a change in the way scientists describe their work. This shift is consistent with the findings of Vinkers *et al.* (2015), who report increased grandeur and positivity of language about results in the biosciences beginning in the 1970s, and escalating more dramatically in the 1990s.

### Experimental technology

Shifts in techniques and methods are also evident when tracking specific words longitudinally over the entire century. Methods crucial to earlier studies of phenotypes which retain their relevance (*e.g.*, cross, Figure 3B) maintain a steady proportion in the literature regardless of decade. In contrast, the trajectories of words like gel or restriction (Figure 3B) associated with particular breakthroughs increase and then decay rapidly. Note that, because we analyze titles and abstracts only, the eventual disappearance of these words does not suggest that these techniques fell out of vogue. Rather, they may have become so commonplace that they are no longer worth mentioning in the abstract.

### Theoretical methodology

**Figure 4:**
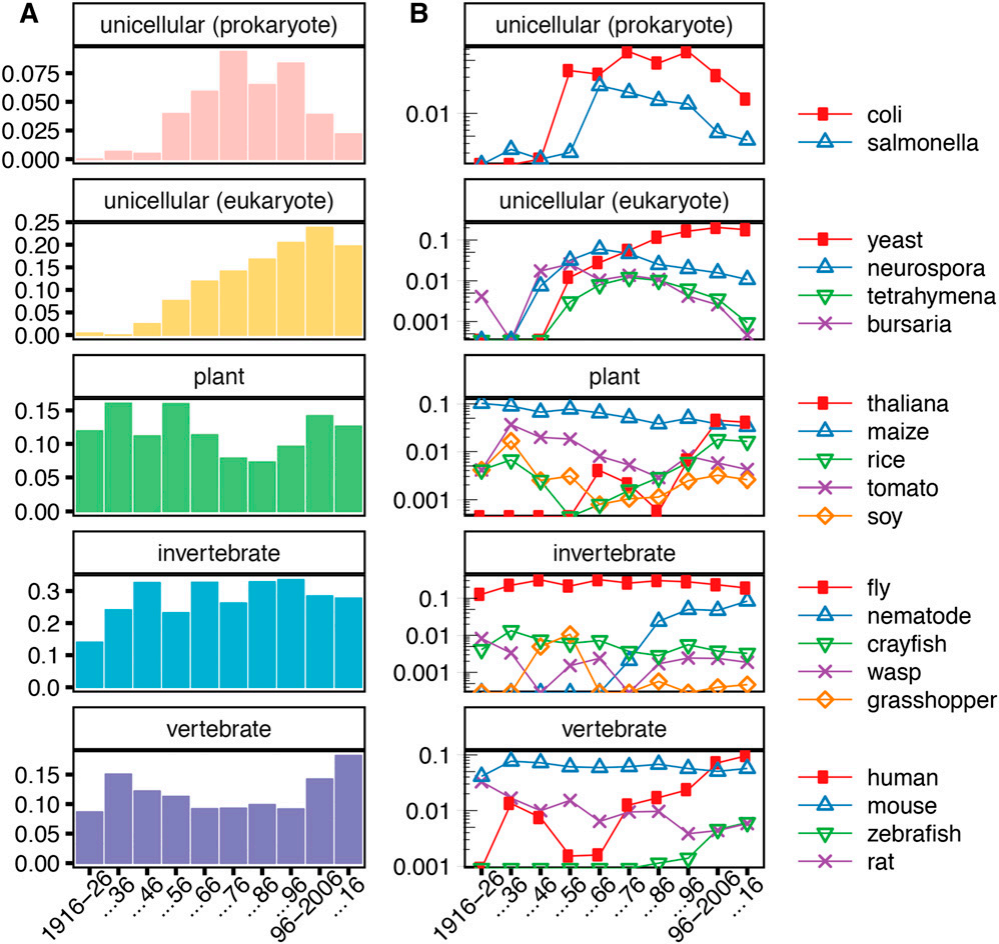
(A) Proportions of all papers that contain at least a single reference to a particular category of organism. (B) A semilog plot of proportions of all papers published in a decade that mention a particular organism. Mentions are defined as at least one usage of a closely associated word; for example, the count for fly includes words such as *Drosophila* and *melanogaster*.

We observed a shift between theory- and experimentation-associated words over the course of the century. There was a decrease in the use of words “theory” and “mathematical” in *GENETICS*, especially during the expansion of experimental techniques utilizing microorganisms from 1940 to 1960 (Figure 3B and Figure 4). However, the birth of scientific computing in the 1970s initiated a comeback for theory, accompanied by a new set of words. This major shift reflects the importance of novel computational and simulation paradigms (Figure 3B, theory), which have expanded in the genomic era, perhaps suggestive of a novel symbiosis between experimental methods and computational analysis.

### Population genetics

Longitudinal tracking of particular words also gives a window into specific subfields in genetics. We investigated the onset and decline of language associated with ideas in population genetics. Though many words exhibit a resurgence or growth after the 1940s, inheritance exhibits consistent decline (Figure 3B). This may be due in part to the increased use of the related but not synonymous heritability (Figure 3B). This hints at the novel context associated with heritability, reflecting changes in the conceptual focus of research about inheritance. The renewal of ideas also likely affects the trajectory of linkage and selection (Figure 3B). Both words fell out of favor rapidly in the earliest decades of *GENETICS*, but as research on inheritance developed further, both experienced a resurgence, unsurprisingly coupled with the word drift (Wright 1931).

We have already remarked on the relationship between experimental methods, lines of investigation, and model organism usage with decade-specific trends. Next, we take a broader look at the scope of organism use. The natural differences between organisms—their visible phenotypes, their life cycles, and other properties—make them amenable to different lines of scientific investigation. Corresponding changes in model organism popularity over shorter timescales in *GENETICS* have been observed previously (Dietrich *et al.* 2014).

We analyzed a subset of organisms that are mentioned in *GENETICS* abstracts or titles and which represent the diversity of organisms included. We extended Dietrich *et al.* (2014) by covering the entire century of publication history of *GENETICS* and by using proportions of papers rather than counts to characterize trends in use. Raw counts cannot be used to characterize century-scale trends in scientific interest due to the substantial expansion in the total number of papers published each year.

### Organisms in *GENETICS*

We categorized trends in the use of broader organism categories as well as subsets of these categories. We manually curated a list of organisms occurring in more than four papers per year and classified them as unicellular prokaryotes, unicellular eukaryotes, plants, invertebrates, or vertebrates. To understand trends in the use of each category of organisms, we used several associated words to attempt comprehensive capture of mentions of organisms in abstracts. For example, we considered any mention of either drosophila, melanogaster, suzukii, pseudoobscura, or simply fly to constitute a mention of fly. In Figure 4, we show these organism-specific trajectories on a semilog plot of the proportion of all papers with at least one mention of a class of organisms (Figure 4A) or a specific organism (Figure 4B).

### Major categories

Multicellular organisms have more stable patterns of use than unicellular model organisms over the course of the century. Specifically, prokaryotic organisms underwent a significant expansion in use and a subsequent contraction. This expansion is also extremely pronounced in yeast and in other unicellular eukaryotes. Most of these contracted substantially by the late 20th century. Yeast is perhaps an exception, although there is a slight drop in the last decade from 20 to 17%.

### Species-specific trends

This organism-specific view elucidates several underlying subtleties of the organism group usage. For example, the stability of the vertebrate group is due almost entirely to the use of mouse (Figure 4B). The vertebrate group’s expansion in the last two decades is primarily attributable to increased mention of humans, owing perhaps to greater interest in or tractability of human genetic studies. Similarly, the stability of the use of invertebrate models is primarily a result of the use of *Drosophila* (Figure 4B).

These trends are stable over the course of the century, but not all multicellular model trends retain such stability. Almost all mentions of plant models at the beginning of the century are attributable to *Zea mays*. Over the course of the century, the proportion of papers studying maize decreased steadily, while other plants were referenced more often (Figure 4B). In the most recent decade, mentions of *A. thaliana* account for almost half of plant mentions over the last decade. While the overall proportion of papers mentioning plants is similar to that at the beginning of the century, suggesting an underlying stability, the species used have changed substantially.

Though many of the unicellular organisms investigated show an extremely similar expansion and decline, there are underlying subtleties (Figure 4B). Though both *Escherichia coli* and *Salmonella* underwent expansions beginning at the same time, the decrease in use of *E. coli* is much more recent than that of *Salmonella*. This decrease may be partly attributed to the utility of *E. coli* in experimental techniques. Like other techniques, it may not be mentioned in the abstract and title, but feature prominently in the *Materials and Methods* section. Other factors, such as changes in journal choice or the growing popularity of yeast as a eukaryotic unicellular genetic model, may also play a role; because of the log plot, yeast’s growth after 1986 is substantial, though it appears less dramatic than the decline of *E. coli* (Figure 4B). As in unicellular prokaryotes, there was an early concerted expansion of unicellular eukaryotes models. But, while mentions of *Neurospora crassa* and *Tetrahymena thermophila* declined substantially, mentions of *Saccharomyces* did not; this genus is mentioned by a majority of papers that study any single-cellular organism in the past decade.

### Discussion

In the past century, there have been massive shifts in the ability of scientists to explore genetic phenomena both experimentally and theoretically, as major advances in biotechnology, engineering, and computation have created new lines of inquiry. *GENETICS*’ long history of leading publication in genetics allows us to illuminate and document this long history. We show trends that are visible by simple examination of text, and these are well-coordinated with major expansion of publication worldwide. As genetics and *GENETICS* continue to develop, it will be fascinating to continue using bibliometrics to characterize the ongoing evolution of genetic research.

## Materials and Methods

### Data access

We retrieved titles and abstracts prior to June 7, 2016 using the Entrez Programming Utilities maintained by the National Center for Biotechnology Information (see *e.g.*, NCBI Resource Coordinators 2016). Specifically, we issued an ESearch query in the PubMed database with the search term 0016-6731[ta], which identifies *GENETICS* articles. The parameters reldate and retmax are set sufficiently large to retrieve PubMed IDs for all articles since 1916. We then used the EFetch utility to download all available text for these items. Though titles are always available, abstracts are only commonly available for papers published after 1946. We used all available text from the EFetch query for our analysis, but note that this may lead to some differences in word frequencies before and after 1946.

### Word enrichment

To identify words whose enrichments grow most strongly in each decade, we considered only words that occurred in at least 100 papers across time. (Due to the low publication rate in the earliest decades, this amounts to only 0.05% of all papers.) For each word, we computed the ratio of appearances in each decade to the total number of appearances over time. Multiple appearances of the word in a single paper were treated as one occurrence to avoid giving weight to lengthier abstracts.

### Model organism mentions

We calculated the percentage of papers mentioning model organisms by manually curating lists of terms referring to each one. (For example, both drosophila and melanogaster are counted as occurrences of fly.) A paper was assigned at most one occurrence of each model organism regardless of how many variations of its name appear in the text. We then calculated the percentage of papers per decade mentioning each word. Notably, as single papers may mention multiple model organisms, it is possible for the sum of proportions across multiple organisms to exceed 100%.

### Visualization

Visualizations were made in ggplot2, using ggmap for maps and the Google Maps Geocoding API for geocoding (Wickham 2009; Kahle and Wickham 2013).
